# Exploring barriers and facilitators to integrating health equity into health and climate change policies in Nepal – a qualitative study among federal level stakeholders

**DOI:** 10.1186/s12913-025-12862-y

**Published:** 2025-05-13

**Authors:** Sudeepa Khanal, Sushil Chandra Baral, Melanie Boeckmann

**Affiliations:** 1 Faculty of Health Sciences, University of Bielefeld, Universitätsstraße 25, Bielefeld, 33615 Germany; 2 HERD International, Saibu Awas Cr-10 Marga, Bhaisepati, Lalitpur, Nepal; 3 Department of Global Health, Institute of Public Health and Nursing Research, University of Bremen, Bremen, Germany

**Keywords:** Climate change and health, Health equity, Climate change policies, Barriers and facilitators, Multisectoral policymaking, Integration, Qualitative study, Nepal

## Abstract

**Background:**

Health is foundational for climate action, and integrating climate and health policies to achieve health equity is widely recognized. While there is a growing global momentum for collaborative health and climate initiatives, more effort is needed to incorporate health equity into national climate policies. Achieving this necessitates identifying both barriers and facilitators of integrated policymaking. This study examines the barriers and facilitators to integrating health equity into climate change-related policies at Nepal’s federal level.

**Methods:**

We interviewed 14 key stakeholders from three major federal ministries, a high-level government entity, and a government partner institution in Nepal, all with diverse roles and responsibilities. To facilitate discussions, we developed an interview guide informed by two policy analysis frameworks: Health Equity Policy Process Analysis Framework and Schlossberg’s Framework of Environmental Justice. Using both inductive and deductive approaches, we identified five key facilitators and four major barriers to integrating health equity in climate change-related policies in Nepal. We present these barriers in relation to WHO’s climate-resilient health systems framework.

**Results:**

A wide array of facilitators was identified, broadly categorized as a) acknowledgement of the need to integrate health equity in climate change policies, b) political leadership, c) global influences, d) established mechanisms and structures in place for collaboration and e) the federal structure. Barriers identified were largely systemic and encompassed a) knowledge gaps, b) ownership and accountability, c) resource constraints: human resources and budget and d) data limitations. Among these, the issue of ownership and accountability emerged as an overarching theme, cutting across all barriers. Similarly, financing and knowledge gaps were identified as significant obstacles to progress.

**Conclusions:**

The findings underscore the need for a more structured approach, with clearly delineated responsibilities to ensure all relevant sectors contribute to the goal of health equity in climate action. Developing well-defined guidelines outlining the roles and responsibilities of different sectors involved in climate action is crucial for fostering ownership and ensuring that health equity is effectively integrated into climate change policies, as well as aiding in resource allocation. We recommend future research to explore the potential role of policy champions within ministries in advocating for and advancing health equity within climate change-related policies.

**Supplementary Information:**

The online version contains supplementary material available at 10.1186/s12913-025-12862-y.

## Background

Climate change significantly impacts health through direct and indirect pathways, exacerbating health equity gaps [1]. Health equity (in the context of climate change) refers to the fair and just distribution of health resources, opportunities, and protections against climate-related risks, ensuring that no group is disproportionately burdened by the adverse health effects of climate change [2]. Likewise, climate vulnerability refers to the extent to which individuals or communities are at risk of adverse climate change impacts and is closely linked to health and social inequities [2]. A population's risk of experiencing climate change-related public health impacts is shaped by social determinants, including disparities in social, economic, and environmental conditions, which affect their capacity to withstand, adapt to, and recover from these challenges [3].

Health sector is crucial in developing climate adaptation policies and fostering climate-resilient development [4, 5]. The United Nations Framework Convention on Climate Change (UNFCCC) [6]—an international environmental treaty aimed at addressing climate change, also recognizes health as a critical sector impacted by climate change. The 26 th United Nations Climate Change Conference of Parties (COP 26), held in 2021 marked a significant milestone by endorsing a health commitment under the UNFCCC [6, 7]. Similarly, the World Health Organization (WHO) has developed an operational framework for climate-resilient health systems to adapt to climate change- related health challenges [8]. It emphasizes the need to incorporate health equity in climate adaptation policies, while prioritizing the needs of vulnerable populations who are most affected by climate change for equitable health outcomes.

In cross-cutting areas such as climate change, integrated policies and coordinated multisectoral actions are crucial to ensure equitable health outcomes [4, 9, 10]. Tangcharoensathien et al. highlight multisectoral initiatives as those that address the interlinkages among the economic, social, and environmental dimensions of sustainable development at local, national, and global levels [11]. Emerging evidence also shows the significant social and health co-benefits of integrated climate change and health policies, demonstrating how addressing climate change can yield multiple benefits [12]. However, despite the growing body of research on the co-benefits of addressing climate and health together, integrated climate and health policies are rare, and integrating health in climate change policymaking in countries has been rather slow with different degrees of attention and fluctuating levels of prioritization among government policymakers [13]. Where policy integration process is complex often influenced by various factors, advancing health equity in climate change and health adaptation in countries, needs to account for contextual barriers and facilitators at multiple socio-ecological levels [14].

Literature is relatively sparse on studies that look at barriers and facilitators to integrating health equity issues in climate change matters, especially in low-middle income countries (LMICs) [15]. A systematic review of reviews identified evidence-based policy, political will and leadership, and institutional arrangements as the three major domains for better integrating health considerations into decision making on climate change mitigation and adaptation in cities [13]. This review consisted of 21 studies, the majority representing high-income countries (HICs), with barriers and facilitators specifically focusing on integration of health as a co-benefit of urban climate policies. Similarly, another study exploring barriers and facilitators at the state level identified funding, state and agency-level prioritization, staff capability and capacity, and political will as factors influencing the readiness for implementation of climate and health activities [15]. WHO has also identified several responsible factors for both inhibiting and maximizing synergies across climate change sectors affecting health [16]. As LMICs experience a great burden of health equity effects of climate change, synthesizing facilitators and barriers for integrating climate change across different areas would be useful for generating insights on common challenges. It is also essential to understand the specific country level facilitators and barriers to better leverage synergies and develop effective integrated climate and health policies [17].

### Context in Nepal

**Fig. 1:**
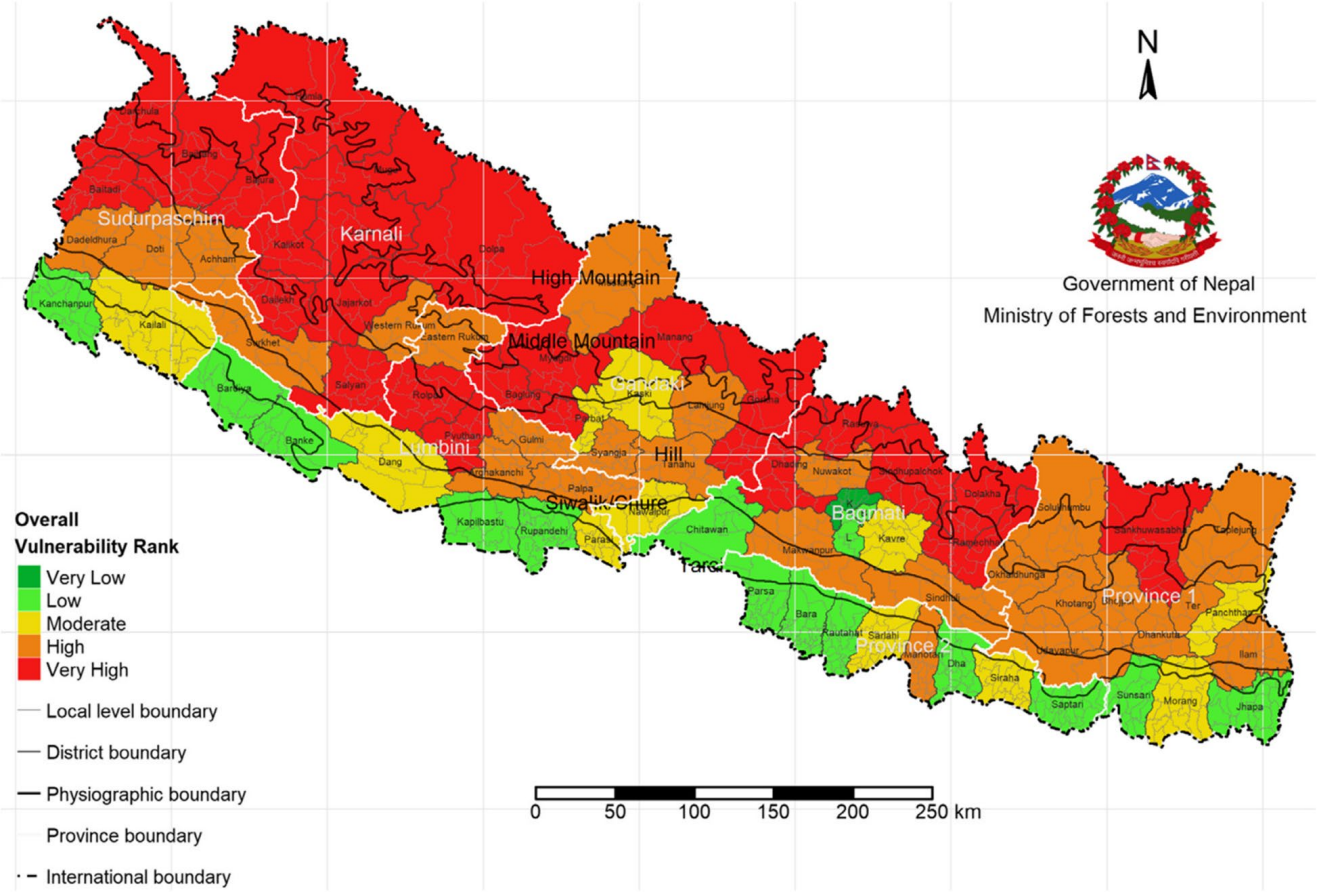
Climate change vulnerability in Nepal. (Source: Ministry of Forest and Environment, Government of Nepal)

Nepal, a federal democratic republic in South Asia, is ranked fourth on the global climate risk index. Despite its low greenhouse gas emissions, diverse topography, complex geology, and varying climate, exposes it to many natural and human-induced hazards causing significant climate vulnerability and challenges [18] (Fig. 1).

Nepal is a signatory to many international protocols related to climate change including the UNFCCC. The Government of Nepal (GoN) [4] has formulated and issued several significant climate change related policy documents, including the Initial National Report to the UNFCCC, the National Adaptation Plan of Action (NAPA - 2007), the Local Adaptation Plan of Action (LAPA - 2010), the REDD Readiness Preparedness Proposal, and the Nepal Climate Change Policy (2011 and 2019) [19].

**Fig. 2:**
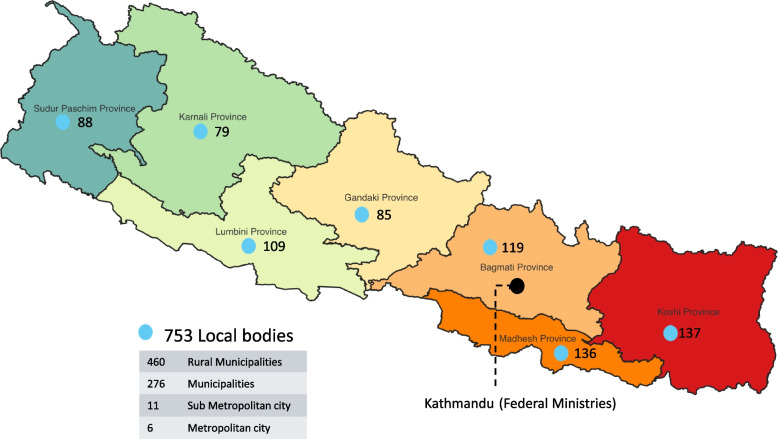
Administrative boundaries of Nepal

Nepal adopted a federal system in 2017, establishing three tiers of government: 753 local, 7 provincial, and one federal government (Fig. 2). The Federal Ministry of Forests and Environment (MoFE) [20] is the focal point for climate change-related issues and its climate change division coordinates with several sectoral ministries for climate change related issues [21]. The GoN has nominated the Ministry of Finance (MoF) [20] as the National Designated Authority and its International Economic Cooperation Coordination Division acts as the contact point for the Green Climate Fund (GCF) [22]. The national climate change policy sets out priority programs in eight thematic and four cross-cutting sectors, with health, drinking water, and sanitation (together) being one of the thematic sectors [23]. The NAPA and LAPA are the main documents guiding the implementation of adaptation programs in the country [18]. In terms of the work in the health sector, the Ministry of Health and Population (MoHP) has successfully developed Health-National Adaptation Plan (H-NAP) [24], however, effective collaboration for integrated planning and joint climate action at different levels remains a challenge.

Nepal Climate Change Policy 2019 provides some insights on general challenges for managing climate change related issues in the country. This includes lack of consistency in comprehending multi-sectoral climate issues among sectoral agencies, insufficient coordination between them, limited research and foundational data on the impacts of climate change and potential losses from climate-induced disasters, failure to integrate climate change concerns into broader development processes, and a shortage of institutional capacity, financial resources, technology, and expertise to tackle these issues effectively [23]. These challenges, however, are not specific to integrating health equity concerning climate change response and there is a lack of essential studies examining facilitators and barriers to policy integration to address this. Such studies would help develop effective, integrated climate and health policies, leverage synergies, and foster effective collaboration to promote health equity in overall climate action.

## Methods

### Aim

This study aimed to explore the barriers and facilitators for integrating health equity in federal health and climate change policies in Nepal.

We used a qualitative case study design employing semi structured interviews with key informants at the federal level ministries to avail in-depth explanations of the barriers and facilitators for integrating health equity into policy measures in climate change.

A semi structured interview guide was developed to understand the barriers and facilitators (Supplementary Material S1).The interview guide was developed combining the components of two policy analysis frameworks: the health equity policy process analysis framework [25] and Schlossberg’s framework of environmental justice [26] for the systematic analysis of facilitators and barriers in various aspects of policy making. The policy process analysis framework is an adapted version of the Walt and Gibson Policy triangle [27] and has been contextually adapted to analyze the “equity approach” in terms of policy determinatives, policy processes and four major equity policy outcomes. Similarly, Schlosberg’s framework of environmental justice is based on three important principles of distribution, recognition and participation, all important components of determining health equity and complements the equity assessment framework to cover important domains for policy analysis [26]. Our previous analysis of key federal documents and health and climate change policies and strategies in Nepal, using Schlosberg's framework, also provided a foundation for this work [28]. The interview guide was reviewed by the research team and a MoHP member, with their feedback incorporated. Additionally, the guide was reflected and adjusted based on interview experiences.

### Study setting and population

The study was carried out in Kathmandu, in the federal ministries of Nepal. Given the indirect linkages of health outcomes with various sectors related to climate change, we chose to limit the data collection to major sectors from four major government entities in Nepal. The participants were mid-to-senior level decision makers from three major ministries: MoHP, MoFE, MoF and the National Planning Commission (NPC). We also included a senior representative from a government partner organization.

### Sampling

To ensure a range of perspectives covering a breadth of experience and explore contradictory perspectives, participants were selected using purposive sampling and snowballing. A list of potential participants was collated based on a review of the organizational organogram. Efforts were made to identify and include female participants where applicable, to ensure gender diversity. However, despite this, challenges were faced in achieving gender representation. A focal person from each MoHP and MoFE was identified and consulted, to approach additional relevant participants. Initial coordination was done via email, phone or in person. The background and purpose of the study was explained and in the case of email and phone coordination, the participants were also sent information sheets and consent forms. Interview guides were provided beforehand, in accordance with participant demands and the interview was scheduled at a mutually convenient time with those willing to participate. No incentives were provided for their participation.

### Data collection and management

One-time, face-to-face semi-structured interviews (average of 1 h) were conducted during October-December 2023. The main author (SK), (a female researcher, with master’s degree in public health and prior experience of conducting national level stakeholders’ interviews in Nepal) collected the data. Previous findings from the content analysis of climate and health policies in Nepal, which assessed health equity considerations, were used to triangulate data during the data collection process [28]. All interviews were conducted in the Nepali language within the ministries’ premises. The interviews were audio-recorded (with consent) and the researcher noted the nonverbal cues and body language of the participants during the interview. The data collected were transcribed and translated to English verbatim by a trained translator who has previously worked with the research team. Translated documents were checked against audio recordings to ensure there was no error in the transcription and translation. The transcripts were pseudonymized removing all the direct identifiers and each participant was given a unique identifier code.

### Data analysis and reporting

Data was analyzed using MAXQDA software. The barriers and facilitators were analyzed both deductively (i.e., coding data according to key elements of the interview guide) and inductively (i.e., eliciting new themes through coding and categorizing of data) using thematic analysis [29] within the broader components of the interview guide. An initial coding frame was developed based on the interview guide. SK independently read through the transcripts, developed codes, and carried out the coding iteratively. The coding process and codes were discussed with senior researcher, MB. Similar codes were merged to synthesize the key facilitators and barriers to integrating health equity and climate change, based on the analysis of participant’s perspectives. The perspectives of officials from different ministries were compared and contrasted to triangulate the findings as necessary and reported. Coding framework details are provided as (Supplementary material S2).

### Ethical considerations

All procedures used for this study was approved by the institutional review board at the Nepal Health Research Council. An ethical clearance was also obtained from the University of Bielefeld, Germany. All participants provided verbal and written consent for participation and sharing of their deidentified data for research purposes. No participants withdrew from the study following informed consent.

## Results

### Participants characteristics

A total of 14 policy makers were interviewed (all male). Despite efforts, the gender distribution was uneven without representation of any female candidate. The age distribution showed a higher proportion of (40–49) and (50–59) age groups with 9 and 5 participants respectively. All except one were government officials. Participants had a minimum of 10 years’ experience working in the government service. The majority of participants held senior to the most senior positions within their institutions, except for one mid-level participant, all in roles requiring engagement in climate change-related decision-making. Table 1 below presents the participants’ profile.

**Table 1:** Participant’s profile

S.N	Education	Gender	Functional role/designation	Age Groups (yrs)	Work experience (years)	Ministry/Institution
1	PhD (Health)	M	Section Chief	40–49	21	MoHP
2	Masters (Statistics)	M	Statistical Officer	40–49	13	MoHP
3	Medical Doctor	M	Division Chief	40–49	10 +	MoHP
4	PhD (Environmental Science)	M	Division Chief	50–59	20 +	NPC
5	Masters (Health)	M	Section Chief	40–49	33	MoHP
6	Masters (Health)	M	Section Chief	40–49	10 +	Multilateral Partner
7	Medical Doctor	M	Section Chief	40–49	10 +	MoHP
8	Masters (Health)	M	Section Chief	40–49	14	MoHP
9	Masters (Environmental Science)	M	Section Chief	50–59	15 +	MoFE
10	PhD (Health)	M	Section Chief	40–49	15 +	MoHP
11	Masters (Environmental Science)	M	Section Chief	50–59	15 +	MoFE
12	PhD (Environmental Science)	M	Division Chief	50–59	20 +	MoFE
13	PhD (Finance)	M	Division Chief	50–59	25 +	Office of the Prime Minister and Council of Ministers (OPMCM)- representing MoF
14	Masters (Environmental Science)	M	Program Director	40–49	12 +	NPC

#### Barriers

- Knowledge gaps

The participants held different preconceptions about the link between climate change and health. We examined their knowledge from two perspectives: general linkage between climate change and health, and the specific linkage between climate change and health equity. Our findings show that there is a low level of knowledge among the participants regarding the connections between climate change and both overall health and health equity. While some participants demonstrated a degree of understanding of the relationship between climate change and health, their comprehension was superficial and limited to certain climate change events or diseases. Many associated health effects of climate change with disasters. The most frequently mentioned connections included health effects due to floods (with or without glacier melting), landslides, and drought. Most participants from the MoHP noted the unusual occurrence of vector-borne diseases as a consequence of climate change. Two participants specifically noted that climate change frequently drives migration, which in turn impacts migratory health.

In terms of the climate change effects on health equity, there was a wide spectrum of responses with only a few participants demonstrating relevant knowledge of the connection. Relatively, more participants from MoHP were aware of the health equity implications of climate change, while for others, knowledge of climate change related health equity effects was vague, and discussions quickly trickled down to climate change and health in general. Conversely, explaining the ongoing rhetoric about climate and health in the country, one stakeholder described how he felt that climate change and its health implications are mere jargon in Nepal.

*“[…]After the COVID pandemic, we are much focused on pandemic preparedness and disaster. That phrase is very popular these days in the health sector, “Building back better”. This and so many others certain jargons are used. Climate change is also one of those jargons. This is a natural process in my opinion” (P1).*

When comparing knowledge among participants across sectors, participants from the MoHP also demonstrated better awareness of the relationship between climate change and health in general. However, three participants struggled to clearly express the connection between climate change and health equity. Many perceived the relationship as a disproportionate impact of disaster events on marginalized groups and populations in geographically vulnerable areas. When asked to provide examples to illustrate their understanding, some participants offered less relevant examples, such as health hazards from unsafe work environments like exposure to x-rays, unfilled sanctioned positions in remote areas affecting health service delivery, and intergenerational equity.

Despite difficulty articulating various pathways connecting climate change and health equity, everyone demonstrated awareness about the interconnected nature of climate change risks across different sectors and its ultimate effect on health. Regardless of their own knowledge, most also noted the lack of understanding among policymakers in this area, emphasizing the need for awareness programs to integrate health equity into climate change-related policies.

b) Ownership and accountability

Different stakeholders had varying perspectives on ownership and accountability for climate change issues in the country, with interview data highlighting these as significant barriers to integration. Overlapping and ambiguous mandates between different ministries were commonly observed among almost all the participants. Without clear consensus on the roles and responsibilities between the MoHP and MoFE, most expressed concerns about the lack of leadership from the other ministry in addressing health equity in climate change matters. All the participants from the MoFE believed that health issues, irrespective of their linkage to climate change, fell under the MoHP’s responsibilities, and they have a limited role to play. They viewed their role primarily as coordinators, coordinating efforts across sectors to address climate change impacts. Along these lines, one participant expressed:

*“[…]Health only thinks about health; education only think about education and……We need to take this linkage in system approach. If we do not go in the system approach, MoHP does not look after climate change at all, and why should they? MoFE is the focal point for climate change in the country. The MoFE on the other hand, who deal with climate change, do not look after health at all. They say the health effects of climate change are MoHP’s responsibility. There is a siloed approach” (P4).*

On the contrary, the perspective of climate change being the responsibility of MoFE, even though its effect ultimately impacts health, was common among MoHP officials. Nearly all of them were of the view that since MoFE has the legal mandate for climate change as a whole, climate change related health concerns also fell under their responsibility. Participants frequently associated policy ownership and responsibility with resource allocation. Linking resources and budget allocation, the majority of the MoHP officials stated that it was unfair to hold MoHP accountable for failing to address health needs arising from the effects of climate change events. They called for MoFE to take the lead on climate change and health issues, with MoHP providing technical expertise as needed. Echoing this concern, one participant highlighted:

*“We should be clear that we need to see the state as a whole. The state has given the entire responsibility of climate change to MOFE. They are legally responsible and have the official mandate to address the issues concerning it (including health effects of climate change). We are not responsible. For example, drinking water can only take responsibility for drinking water. Sometimes, confusion is that we (health sector) are looking at other sectors as well. I think we need to make everyone, and our partners, understand this…. We should not be responsible for everything. If we take on responsibilities which are beyond health sector, then our performance will always be low….” (P7).*

The lack of clarity was evident not only between the two-line ministries but also within the broader governing and advisory institutions. Within the government, the NPC was identified as the leading agency on climate change issues (particularly by MoFE officials), offering overall policy guidance to the MoFE. However, confusion also persisted among NPC officials regarding who should take responsibility for advancing health and climate change matters. Furthermore, they were uncertain about their own role in facilitating this integration. In this respect, as one participant noted:

*“[…]The concerned ministry develops the policy, and climate change policy is just a framework. The stakeholders of the policy may be health, education, physical infrastructure, or others…My understanding is that “whatever the policy is there, if it is related to other sectoral ministries, they have to develop their own program and budget” (P13).*

Yet another participant recounted:

*“Role of NPC is also crucial because it is responsible for providing guidance, directives, and feedback for policy development. An internal meeting at NPC is organized to discuss the draft and provide feedback. The division concerned looks for areas of improvement and things to be added to the draft. Since we are general bureaucrats, we are not trained to analyze the linkage between climate change and health, its impact and situation. We only think like a layman. While reviewing the draft, we look at it in a general way. It is certain that there are certain lapses in our analysis. We normally go through the draft and say whether it is okay. Until you came here, I don’t think we even realized that health topics are not covered much or are missing in the climate change policy. We need to first realize how climate change will impact children and senior citizens. We then need to analyze, diagnose, and track the most vulnerable population segments” (P4).*

Despite everyone discussing the lack of clarity in roles and responsibilities as a barrier to integration, one participant explicitly stated that clarifying these roles and responsibilities is the only way forward to mainstream health equity in climate change policies.

*“[…]It is important that we clearly define roles and responsibilities. The notion of supremacy of one sector over another does not always work. Coordination should always be mutual. Both parties need to coordinate. So, if we could make the roles and responsibilities clear, then there would be no big issue. In federalism, although the role is clear, it has just touched superficially. So, it seems vague… The work that cannot be done by MoFE should be provided to concerned sectors. Things are just written up. If we could make the roles clear, there would be no problem regarding coordination. But if we cannot make the roles clear, none of the mechanisms work” (P13).*

Another participant pointed out an interesting aspect, suggesting that ownership and capacity of institutions depend not on systems or mechanisms, but on individuals holding the key positions. This idea was consistently reflected in the data, giving the impression that the current integration of climate change and health equity in Nepal relies on a few key stakeholders, or"policy champions". These stakeholders were frequently referenced in discussions on the topic across the sectors, with all participants from MoHP and three from other ministries identifying specific individuals as experts in climate change and health.

c) Resource constraints: human resource and budget

All the participants voiced inadequate financing for health-related climate change programs as one of the most significant barriers to integrating climate change and health equity. Inadequate financial resources were a common problem pertaining to both the health and environmental ministries. According to both the MoFE and MoHP, neither of them had financial flexibility to incorporate climate and health equity related activities in their regular programs. In this aspect, MoHP perceived themselves as particularly disadvantaged because despite the ultimate (direct or indirect) impacts of climate change on the health sector, there was no budget provision for addressing the issue. Lack of resources compounded by competing program priorities were seen to negatively impact the uptake of climate change and health agenda for MoHP. In the absence of budgetary provisions specifically for climate change and health related activities, they clearly expressed expectations from the MoFE to develop integrated climate change and health program and policies.

*“[…]NPC makes all our budget code. There is a climate change budget code. And health has zero budget for climate change. Because there is a budget ceiling in health and health does not want to cut off budget from other existing programs. But in the other ministries, budget is provided if we want to address climate change” (P10).*

Consequently, under these circumstances, almost all the officials from both the ministries reported the need to rely on external funding or support from the external development partners (EDPs) to carry out activities related to climate change and health. However, they noted that the number of externally financed projects and partners in the nexus of climate change and health in Nepal to be limited. Inadequate access to various climate finance mechanisms was highlighted as a barrier by the majority of the participants. The complicated process for applying for the climate financing mechanism [such as GCF, Global Environmental Facility (GEF) fund among others] coupled with stringent eligibility criteria for application were repeatedly iterated as a hindrance for accessing climate finance.

In terms of human resources, across all entities other than MoHP, there was an understanding that if any climate and health– related activities had to happen, it had to go through the MoHP’s structures. In this regard, officials from the MoFE and NPC appreciated the MoHP’s mechanisms and their access to grassroot communities through health post and community health volunteers. However, not all the MoHP participants agreed that MoHP should take a leading advocacy role in integrating health equity into climate change planning across sectors. Some of them also underscored the increasing strain on health facilities and staff if all interventions and accountability related to climate change and health were assigned solely to the MoHP. Staffing constraints and frequent staff turnover were also mentioned as factors impeding the provision of equitable health services in climate-change related programs by some the MoHP officials.

Two participants highlighted the lack of technical capacity as a key challenge and mentioned the need for awareness raising and technical training of healthcare providers in matters related to climate change and health equity. Regarding training initiatives related to climate change and health integration, accounts of participants varied, with some mentioning federal-level training and others noting training aimed at health workers in hilly and mountainous regions focusing on vector-borne disease diagnosis, treatment, and management. These trainings were predominantly mentioned by MoHP participants, who emphasized the need for further targeted capacity building activities to MoHP officials as well as to stakeholders in related sectors, particularly MoFE, to enhance awareness. In contrast, MoFE officials placed less emphasis on human resource provision or specific training needs, viewing it as the role of the MoHP.

d) Data limitations and their use for decision making

Although participants’ acknowledged the public health impact of climate change from both local and global studies, many expressed concerns over the insufficient up-to-date data to inform policy development and reported challenges to access information for decision making. This data gap was often highlighted as a significant bottleneck and a major barrier to understanding the health effects of climate change, thereby informing integrated policies. Inconsistencies were reported regarding efforts made to prioritize health equity in climate change decision making in the country. The understanding of the country's current stance and progress in integrating health equity into climate change-related policies was more individualized than institutionalized.

Despite having conducted health and vulnerability assessments and developed H-NAP in the country, most participants were unaware of its existence. They expressed the need for health specific vulnerability data to promote efficient policies and plans. When asked about the available data sources for climate change-related health vulnerabilities, participants gave varying responses, often referring to basic national-level sources such as census data and household surveys. While most participants pointed out that current policies and planning relied on limited data, few noted that not all available research evidence was being utilized. Nevertheless, there was a general agreement that increased research and vulnerability data could serve as a starting point for integrating health equity into climate change policies.

In this context, one participant mentioned that the Nepal Health Research Council (NHRC) had set up a data center as a repository for climate change-related data in the country. However, the initiative was not sustained, primarily due to funding constraints. Notably, only one participant was aware of this, and no others reported it. Three other participants (two from MoHP and one from MoFE) reported having signed an MOU with the Department of Hydrology and Meteorology (DHM) and MoHP to model some climate-sensitive health indicators and for climate change-related disease surveillance. One participant further emphasized this, stating:

*“Main problem is that we have data in our pockets, but we do not have a sharing mechanism. HMIS data is limited to making annual reports. It is not used further. DHM data is also not used further. We don’t have a system to inter-collate, inter-link and model those data. That needs to be done. At least there has been a bit of progress and there is a MoU between the department of health services and department of hydrology and meteorology to share data. Otherwise, we needed to buy data. But although it is signed, it has not been materialized, yet” (P10).*

#### Facilitators

- Acknowledgement of the need to integrate health equity and climate change

Climate change was widely recognized as a public health concern and a problem worthy of attention. There was an increased awareness that climate change generally causes adverse health effects. Almost all the participants across sectors unanimously agreed that the health sector and marginalized populations ultimately bear the brunt of climate change impacts, prompting a desire for integrated climate and health policies. Reasons cited for this acknowledgement included experiencing changing disease patterns, decreased agricultural productivity, increased public awareness, and a young workforce with academic exposure to the issue, among others.

Even though sectoral differences were observed among ministries in terms of health equity considerations, only four participants from the health ministry emphasized integration specifically in terms of health equity. Participants from other ministries also mentioned health equity but often used the term interchangeably with the broader concept of incorporating health into climate-related policies. All participants agreed that addressing climate change and its effects requires inter-ministerial collaboration, rather than being the responsibility of a single ministry. The call for integrated approaches to tackle these challenges was evident, with a recurring recommendation across all sectors to develop and implement integrated policies for climate and health. As one participant noted:

*“We only co-ordinate on the issues regarding climate change but it is everyone’s concerned subject. It cannot be addressed if everyone doesn’t do things from their sides. We cannot say any specific sectoral ministry is responsible for climate change matters” (P9).*

Another participant said:

*Climate change is a global negotiation. People here think that in COP, developed countries provide funds in dollars and we go there to collect it. But that’s not the truth … All sectors should understand this. The climate crisis is ongoing… We need to predict and start preparing for that” (P11).*

b) Role of political leadership

There were mixed opinions on political leadership. Most participants reported good political leadership, particularly citing the example of the recent COP 28 conference, which was attended by the prime minister along with the minister of MoHP. While many believed that their participation was motivated by genuine interest in the country's welfare, few participants considered it a publicity stunt. Some participants expressed skepticism, believing that the priority given to climate change and health in the country is primarily influenced by donor interests, and that the government does not prioritize its own agenda in negotiations. One of the participants elaborated:

*“In any country, everyone’s level of understanding is not the same, but slowly the understanding level is increasing in general. The perception is being changed. Each sector has been aware that we should take the lead in our respective areas. Even the political parties are aware that climate change governs geopolitics now. So, the overall understanding level has increased but it is not the same for everyone” (P11).*

Regardless of the reasons behind the growing interest in integrating climate change and health among the policy makers and leaders, most participants believed that senior government officials are willing to address the issues of climate change and health (equity), even if it means relying on EDPs for financial assistance to implement these initiatives. One participant pointed to the “young health workforce” as a key factor driving the inclusion of climate change and health agenda in policies. Having studied these issues during their education, they are more receptive and advocate for integrating health equity concerns into climate change policies. In this context, one participant made the following narration:

*“H-NAP was not developed just by willingness of EDPs. There are synergies between both. The government had willingness and EDPs had the resources. All those things made this possible, the official government structures, need willingness and the resources of development partners…….When young leaders like us entered in the health ministry, (we bring new issues) …You and I have just completed the public health study. Had there still been people in the system who had completed their masters, public health, or global health some 20 −15 years back, issues of climate change would not have been there…That has contributed” (P1).*

c) Global influences

It was evident that the climate change efforts in the country are largely influenced by the global context and policy formulation is driven mostly by international efforts. Participants recognized the impacts of global events and initiatives on national policy development. Majority of the participants stated that the primary reason for initiating the talks around climate change and health in Nepal is due to the international commitments that the country has made, and their reporting obligations. They mentioned that the need to submit periodic progress reports to various international agencies served as a reminder for them to work in this area.

One of the high-ranking participants stated:

*“[…]may be (the policies are developed) due to demonstration effect. If you do not go with the global agenda, you feel that you are falling behind. [Laughs] Second, they may have written without understanding. Why everyone does that because they think they should not be behind any global agenda… It will be easy to implement if the policy is spelled out based on the evidence. Otherwise, you just write it, and you do not have any idea on how to implement it… that’s the case in Nepal” (P13).*

Another participant highlighted:

*“I talked about resources, influences, and global advocacy. In our context such global advocacy influences the most. One of the reasons for health and climate change getting onto the agenda is due to global evidence and advocacy. Also, some research evidence has influenced this” (P8).*

The (then) upcoming COP 28 was also identified as one of the main reasons for increasing awareness about the need for integration within the NPC and MoFE. Participants were aware that health was a focus area of COP 28, as the minister of health (MoHP) along with other delegates from MoFE were to accompany the prime minister to the COP 28 event.

d) Established mechanisms and structures for collaboration in place

All the participants acknowledged the existing legal and institutional framework for collaboration in the country. They noted awareness about the MoFE identification of eight ministries relevant to climate change, each with dedicated thematic working groups. They demonstrated awareness of these groups being coordinated under a joint committee led by MoFE. Prominent structures mentioned by the participants at the federal level included the Interministerial Climate Change Coordination Committee, the Ministerial Development Action Committee (MDAC) chaired by sectoral ministries including health, and the National Development Action Committee (NDAC) chaired by the Prime Minister under the coordination of NPC. Additionally, they reported the existence of a National Development Council led by the Prime Minister, with members from federal and provincial governments. Similarly, they acknowledged that provincial Climate Change Coordination Committees had been established at the provincial level to coordinate the activities of various sectors as per the directives of NAPA. Several relevant structures within MoHP and MoFE were noted for coordinating activities within different sectors at all levels for environmental health-related initiatives.

Disaster response was frequently cited in discussions about climate change and health with several participants mentioning it. The participants mentioned that the Ministry of Home Affairs (MoHA) serves as the central federal ministry, supported by Chief District Offices in each district and the National Disaster Risk Reduction and Management Council (NDRRMC) in each municipality. The roles of Health Emergency Operation Centre (HEOC) and Provincial HEOCs were specifically noted for supporting NDRRMC in rescue operations, shelter management, and food distribution. However, despite these platforms, some participants expressed concerns about functional overlap and confusion over roles and responsibilities between the ministries and these committees, leading to uneven prioritization of health in climate change matters.

*“It is good that MoFE is looking after climate change as it is its area. Climate change is related to forests and water resources. But I think inter-sectorial co-ordination, co-ordination between government and non-government and co-ordination with other private sectors is lacking… Because of the co-ordination issues, health issues have been reflected less in the climate change related documents. MoFE focuses more on its sub sectors and area. Other sectors or areas have to come there” (P14).*

e) The federal structure

Federalism was viewed as an opportunity by most participants to integrate health equity in climate change policies, highlighting the autonomy of provinces and local governments in planning and policy formulation. They appreciated how decentralization has enabled provincial and local levels to develop and prioritize their own policies and programs. However, they noted that none of them were known to have incorporated health issues into their climate change planning, including the highly vulnerable provinces and municipalities. Inadequate awareness about climate change and health linkages, unclear roles and responsibilities across different ministries and tiers, no dedicated department at the local level for climate change and health, limited data and (financial) resources, other (tangible) competing priorities were frequently mentioned reasons for this. A few participants also cited limited budget allocations as an issue at the local levels and expressed concerns about the federal level distributing equalization grants not based on data, but often in an ad hoc manner.

*“We are in the federal context. Both the province and local level are autonomous in this context. They are provided with a certain unconditional grant which they can decide themselves about how to utilize. They can utilize it on the basis of their context, needs and vulnerability. That is a good aspect…The local level itself works to address the health problems linked with climatic variables” (P1).*

There was an emphasis on the need to raise awareness at the provincial and local levels about the linkages between climate change and health and health equity, and to encourage the establishment of separate divisions/sections to address these issues effectively. Additionally, a significant gap in policy communication was observed between the federal and sub-national levels. For instance, the formulation of H-NAP was not effectively communicated to sub-national governments, resulting in its limited incorporation into their annual planning and budgeting processes.

*“I think awareness level is very low in provincial and local levels. Wherever LAPA is implemented, they have focused on agriculture. I don’t think LAPA has focused on health. The general perception at the province and local level is that development means construction of roads and bridges. LAPA focuses more on agriculture, drinking water, irrigation, *etc*.” (P12).*

## Discussion

This study is among the first to examine the barriers and facilitators for the integration of health equity in climate change-related policies in Nepal. Our findings show that integrating health equity in climate change policy discourse in Nepal is complex. Specifically, focusing on climate change and health, our study complements the most common barriers and facilitators for developing multisector policies [30, 31].

While most identified barriers were systemic, the facilitators encompassed broader structural factors, such as national structures, global influences, and positive political will. The study emphasizes that although there is widespread recognition at the federal level of the need to incorporate health equity into climate policies, achieving meaningful integration remains difficult. The primary challenge to integrating climate change and health equity in Nepal's policies stem from issues related to ownership and accountability. Ambiguity regarding the roles of different sectors in developing integrated policies, coupled with limited guidance from the NPC, hampers efforts, leading to a lack of ownership among key ministries. While some ministries view the health impacts of climate change as falling under the MoHP's mandate, this perspective is not universally shared within the MoHP itself. This lack of ownership and accountability as a barrier to cross-cutting policy issues is not new and has been documented as a major hindrance for policy integration in multisectoral policy making [32]. Additionally, even though other barriers such as financial and human resource constraints are important barriers that warrant further exploration, as Madrigano et al. suggest, insufficient funding is often compounded by the perception that climate change is primarily an "environmental" issue. This narrow view tends to overlook the health risks of climate change and neglects system-based approaches [33]. This situation is also evident in Nepal. Therefore, it is extremely important to address this alongside, if not prior to other institutional barriers.

Another notable barrier identified that requires immediate attention is the insufficient awareness of climate-health (equity) linkages among the stakeholders, particularly those outside the health sector. Research indicates that knowledgeable and informed health professionals are better equipped to convey health risks of climate change and benefits of adaptation [34]. Moreover, the self-efficacy and confidence of health professionals in implementing climate and health initiatives play a crucial role in promoting effective policies [15]. It is therefore important to establish mechanisms for learning and knowledge sharing between ministries to increase awareness of these linkages and support advocacy for policy integration. Other institutional barriers such as data scarcity as well as underutilization of existing data, and limited capacity for evidence-based policymaking, further hinder progress.

Despite these barriers, current policies in Nepal are well-aligned with global efforts on climate action, with the country adopting components of the NAP and meeting the requirements of the Paris Agreement [23]. The primary facilitator identified for integration was the widespread positive acknowledgement of the need to integrate climate change and health policies. Underlying this perception was a consistent recognition of the need to follow the global agenda. Globally, in recent years, the climate crisis has increasingly been framed as a health crisis [35, 36]. Incorporating health into key events like COP has the potential to trigger countries to initiate integrated actions on climate change. This global discourse has also influenced Nepal, prompting the inclusion of health equity considerations into climate-related policies at the country level. One example of this is the development of the H-NAP which marked an early effort to integrate climate change and health [37]. While this was a promising step towards concerted action for integrating health equity, its progress has been slow.

Several other existing factors were noted to play a facilitatory role for integration. A key factor highlighted was the positive political will among the politicians, which is crucial for driving such integration efforts forward. Numerous studies have consistently demonstrated a significant connection between politics and policy outcomes [38, 39]. Though our study highlights political leadership as a positive force in climate and health integration efforts, its translation into tangible actions remains questionable with no evidence of this integration in the national budget or financial provisions of the MoHP or the MoFE [40, 41]. Nevertheless, having high-level politicians on board, regardless of the underlying motivations, can be considered a strength.

Another advantage that Nepal has is few stakeholders already working relentlessly to include health equity considerations in climate change policies. These individuals also seem to have gained recognition as experts in the intersection of climate change and health across various sectors. Although evidence on the influence of these “policy champions” on policy uptake is inconsistent and challenging to assess, few research supports this approach [42]. Taking a deeper look at who these identified “policy champions” within the systems are, and how they can be better supported and empowered to drive policy integration that advances health equity within the climate change initiatives would be beneficial. Additionally, various existing structures and mechanisms (in the context of federalism) provides a crucial context for integration. Both national and local policies could benefit from existing multisectoral platforms that could be used to facilitate discussions on climate change issues and contribute to integrated policymaking.

The findings from this study concur with those of a systematic review on the barriers and facilitators for integrating public health benefits into urban climate policy, which identified political will and leadership, institutional arrangements and evidence-based practice as major domains influencing integration of climate change and health policies [13]. However, unlike this review, which also explores challenges in utilizing evidence for policymaking, our study highlights a strong demand among policymakers for more specific health vulnerability data, though it does not examine the potential impact of such data on decision-making in detail.

### Closing the gap for developing climate resilient health system in Nepal

**Fig. 3:**
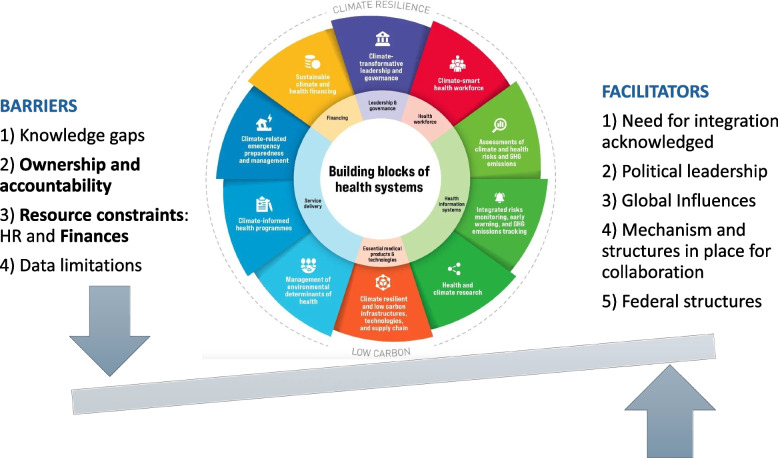
Barriers and facilitators for integrating health equity into climate change related policies in Nepal (Adapted from WHO [8])

The study identifies challenges across all ten components of building climate-resilient health systems [8],with governance, leadership and, climate and health financing emerging as particularly crucial areas (Fig. 3). While the WHO has identified ministries of health (in respective countries) as the lead agency for the developing H-NAP [8], the lack of ownership and policy coherence among various ministries remains a significant issue. Although sub-optimally explored to date, it is plausible that similar barriers and facilitators would appear in other LMICs with comparable contexts.

The challenges of multisectoral integration in health are well established, yet there is currently limited evidence on effective strategies to overcome these barriers in the context of climate change and health equity, particularly in LMICs [31]. Furthermore, empirical evidence and experience to support strategies for addressing the issues of ownership in climate change and health is relatively limited [4]. A scoping review conducted by Mosadeghrad et al. [43], identified 87 interventions for strengthening a climate-resilient health systems, grouping them into WHO’s six categories of building blocks of health systems [43]. Of these, 16 specific interventions were proposed for governance and leadership. These included developing national adaptation plans, engaging governments, refining health sector regulations, creating comprehensive policies, raising awareness among healthcare leaders, designing crisis leadership models, collaborating with climate change institutions, empowering local stakeholders, enhancing collaboration, decentralizing management, and designing a climate change framework. The review stresses the importance of considering external factors- such as political, economic, social, environmental, and legal elements, when addressing the adverse impacts of climate change on health systems. While these findings are highly relevant to Nepal, they do not provide further clarification or guidance on how to implement these interventions to create transformative governance in the context of climate change and health in a country. Addressing emerging climate change-related health equity issues requires more focused discussion on how countries (like Nepal) can respond within their given context.

Though our study did not explore integration at subnational levels, these levels play a critical role in preventing and managing the health effects of climate change [44]. While effective national policies are essential in a federal context, it is equally important to harmonize global and national evidence with local solutions to ensure that policies are contextually relevant while remaining aligned with international standards. Achieving this requires collaborative global and national efforts to strengthen policy support for integrating health equity into climate change-related policies. To facilitate a country-driven process, it is essential that policies are inclusive, evidence based, and reflective of diverse perspectives, while building on existing national efforts to integrate health into climate change responses [45]. Moreover, there are broader questions regarding the role of the global community and organizations—not only in advocating for health equity within climate agendas but also in providing support and guidance on adapting global frameworks to address the distinct needs of local populations.

The need for increased international cooperation, better access to financial resources—particularly for vulnerable regions, sectors, and groups—inclusive governance, and coordinated policies has also been proposed by the Intergovernmental Panel on Climate Change [46] for climate-resilient development [46] and reiterated in the COP 28 [37]. In contexts where acquiring sufficient internal resources and financial support is difficult, improving access to climate finance would enable the national government to develop and implement initiatives aligned with national priorities.

### Limitations

This study had a few limitations. First and foremost, all relevant participants included were male. This selection wasn’t deliberate and reflects the gender representation in decision-making positions in Nepal. The underrepresentation of women in leadership and policy development roles is a well-recognized issue in the country. Despite our efforts to include a female participant from a relevant government position to ensure gender diversity, we were unable to secure her participation due to her unavailability during the study period. This limitation underscores the challenges of achieving gender balance in specific sectors and highlights the ongoing need for initiatives to promote women's involvement in decision-making roles. Secondly, the study focused exclusively on participants from the federal level. Although the federal-level stakeholder’s perspectives on integrating health equity at the provincial and local levels were identified, including participants from these levels could have provided additional insights and strengthened data triangulation. However, the study aimed to explore barriers and facilitators at the federal level. In many federal countries, national policies play a crucial role in guiding subnational policies, with national planning serving as a framework for states to develop detailed policies and plans. Nonetheless, we believe the general barriers and facilitators identified at the federal level are likely relevant to provincial and local governments as well. Conducting similar studies at these levels would further deepen understanding in this area. Thirdly, since most of our participants were senior-level policymakers, securing time for an interview was a challenge. Additionally, there was a time constraint with some participants, and the interviews had to be completed within a certain period. This was anticipated and consistent with our expectations. Therefore, in time-constrained interviews, we focused on the major questions from the interview guide. Finally, although we wanted to recruit participants from the MoF, we couldn’t schedule an interview. However, we managed to recruit a high-level official who had an overview of the climate change budgeting and financing system.

#### Researcher reflexivity

(Supplementary material S3).

## Conclusion

This study highlights the complexities of climate change policy and the integration of health equity within it, identifying key systemic and structural barriers and facilitators in Nepal. It clearly points out that addressing ownership and accountability is essential for integrating health equity into climate change initiatives, supporting resource allocation and overcoming other systemic challenges. Establishing a clear framework that defines the roles and responsibilities of each ministry is crucial to ensure accountability and foster effective collaboration. The study also emphasizes the need to enhance stakeholders'understanding of the links between climate change, health, and health equity, while also addressing other long-standing challenges such as financial resource limitations and data gaps. The findings advocate for a context-specific strategy to promote intersectoral collaboration to develop policies that are not only aligned with global standards but also deeply rooted in the local context. Additionally, it calls for further research on the role of"policy champions"in advancing the climate and health agenda. Overall, the insights from this study can serve as a valuable resource in formulating climate change and health-related plans and policies, contributing to the development of a climate-resilient health system at the national level and supporting global efforts for multisectoral action to ensure no one is left behind.

## Supplementary Information

Supplementary Material 1.

Supplementary Material 2.

Supplementary Material 3.

## Data Availability

The datasets (interview transcripts) generated and analyzed during the current study are not publicly available to protect the anonymity of participants within the local authority. However, unidentifiable data can be made available from the corresponding author upon reasonable request.

## Abbreviations

EDPs: External Development Partners
GoN: Government of Nepal
COP: Conference of Parties
GCF: Green Climate Fund
HICs: High Income Countries
H-NAP: Health National Adaptation Plan
LAPA: Local Adaptation Plan of Action
LMICs: Low- and Middle-Income Countries
MoF: Ministry of Finance
MoFE: Ministry of Forest and Environment
MoHP: Ministry of Health and Population
NAPA: National Adaptation Plan of Action
NDRRMC: National Disaster Risk Reduction and Management Council
NPC: National Planning Commission
WHO: World Health Organization
UNFCCC: United Nations Framework Convention on Climate Change
