# Supplementary material for: Exploring barriers and facilitators to integrating health equity into health and climate change policies in Nepal – a qualitative study among federal level stakeholders
Source: BMC Health Serv Res. 2025 May 13;25:687. doi: 10.1186/s12913-025-12862-y (PMC12070595; doi:10.1186/s12913-025-12862-y)
Supplement: Supplementary file 1 — Supplementary Material 1. [file 12913_2025_12862_MOESM1_ESM.docx]

**Title of the study: Exploring barriers and facilitators to integrating health equity into health and climate change policies in Nepal – a qualitative study among federal level stakeholders**

**In-depth Interview guideline for policy makers on Climate Change and Health Equity**

Respondent name

Presented gender

Education background/level

Respondent job title

Respondent contact information

Respondent email address

**Integration of health equity in climate change related policies will be explored from the perspective of environmental justice principles. The barriers and facilitators will be analyzed with regard to recognition, distribution and participation for each of the policy determinatives, policy processes and policy outcomes.**

**Brief statement about purpose of interview, ethics, right to withdrawal, recording and taking notes**

**Warm up questions**

1. Tell us something about your job responsibilities? How long have you been in this position?
2. In your opinion, how are climate change and health related?

- What do you think are the health implications of climate change?

**Policy determinatives**

1. Context/evidence/ nature of health equity issue
2. How do you understand health equity in relation to climate change?
3. How is health equity understood in terms of climate change in Nepal? (by different sectors/ beyond disasters) – explore if understood from the perspectives of SDoH, Environmental justice, social vulnerability, environmental burden, health vulnerability?
4. How do climate change related policies address health equity?

- To what extent do you think the (health) equity aspects are included in the (climate change related) polices? (How)
- How are health equity issues defined in the policies? (Is there a clearly defined problem?)
- In what context is equity/equities or inequity/inequities mentioned?
- Why did the policy include it? Or are there any factors (political /donors/ other) to get the issue (of Health Equity and Climate Change) on the agenda of MoHP/ MoFE/MoF others? -formal structures for collaboration, resources, actors, policies, commitments to international conventions, evidence on impacts, issue of social/environmental /climate justice etc.- both positive and negative?

1. Have there been any key factors or efforts to include health equity in the policies? -**in terms of the context- influencing/ inhibiting factors**?
2. What do you as an organization need to be able to focus on health equity in climate change related policy?

**Policy Processes**

Agenda setting/policy development/policy implementation and evaluation AND Actors (Role/Agenda/Resources)

1. Can you tell me a bit about the process of creating a climate change policy in your institution?

- Who starts the process, who leads, who gives input, what are the steps to follow, is there a protocol, how long does it take, what are your goals with these policies, is improving health equity an explicit goal?)

1. How are the health equity related problem/issues identified?
2. Can you tell me a bit more about how different populations are considered in the climate change policy process?

- How are risks to different populations identified?
- How are differences in income, education, ethnicity, gender, for children or adults considered?
- What evidence is available in relation to HE in CC? To what extent are the reports considered while developing policies? -what are the difficulties? (Reminder: VRA of MoHP identifies the risk and the HE impacts to large extent)
- Does the word health equity ever come up or are different terms used when talking about disadvantaged populations?

1. Who are the key actors and what are their roles in the policy making process? - role of federal government, External Development Partners, others?
2. How do different actors work together? Can you please describe the process of working together across sectors on a specific policy?

- Who takes the lead, engagement with other ministries? Are appropriate and necessary individuals/departments/ministries involved in the process?
- Involvement of target population/vulnerable communities in the decision-making process? Any mechanism to ensure their participation?
- How are the decision agreed on?
- Who implements what? How is this decided?

1. What role do you think governance structures/how the departments are set up/ structures and resources play in designing CC and climate change related health policies?

- How do the governance structures in the relevant ministries/department enable or challenge effective policy development to include health equity issues?

1. How are the resources managed? Provision for budget allocation? (Who provides budget for what/ any provision to include the vulnerable groups?)

- Following the budget code, access to green climate fund, funds from the external development partners, off red book budget etc
- How are the policy (actions) decided?

1. Are there any specific processes/measures to ensure incorporation of health equity in climate change policies (or health policies related to Climate Change) **in terms of the processes?** What are they?
2. What do you consider the main facilitators / enablers to this process? - (example- constitution, Health Policy, national Climate change policy, Health in all policy approach?)
3. What do you think are the main challenges to integrating health equity in climate change related policy development processes?

**Policy Outcomes**

1. How are the health equity in climate change policy outcomes measured?

- Are there any outcomes related to Inclusion of SDOH, Participatory policy process, equitable, timely, affordable healthcare services for vulnerable groups/evidence generation for vulnerable groups? - regularity and reliability for recording, analyzing, reporting these? Facilitators and barriers for this?

1. How are the policy/implementation strategies monitored or evaluated? Who does it, how frequently, accountability?
2. Are there clear, credible indicators to understand health equity issues that demonstrate the severity of the problem and that can be used to monitor progress- for Monitoring and evaluation?
3. What are the barriers for implementation, monitoring and evaluation of these policies?
4. What are the facilitators to implementation, monitoring and evaluation of these policies.

**Concluding Questions**

In your opinion what can be done to advance HE in CC related policies?

- build understanding and commitment to health equity in terms of efforts to address effects of climate change?
- Develop organizational knowledge and skills to advance health equity in relation to climate change?
- Align programs and resources with an organizational commitment to health equity?
- Work in true partnership across the community and sectors to advance health equity?
- Improve data collection, analysis and use of data to advance health equity?
- Advance health equity at the policy level?
- What recommendations do you have for future efforts?

Is there anything else that you would like to add?
