# Supplementary material for: Exploring barriers and facilitators to integrating health equity into health and climate change policies in Nepal – a qualitative study among federal level stakeholders
Source: BMC Health Serv Res. 2025 May 13;25:687. doi: 10.1186/s12913-025-12862-y (PMC12070595; doi:10.1186/s12913-025-12862-y)
Supplement: Supplementary file 3 — Supplementary Material 3. [file 12913_2025_12862_MOESM3_ESM.docx]

### Researcher’s reflexivity

I (SK), one of the researchers, involved in collecting the primary data has previous experience working in Nepal's health sector, including conducting interviews for policy analysis in another health domain. I have also worked with one of the Ministry of Health and Population’s (MoHP) partner organizations in Nepal, supporting the government in implementing and advocating for evidence-based decision-making. My professional experience in public health in Nepal inspired me to explore the integration of health equity into climate change policies in Nepal more deeply.

Since our study involved government ranking officials, our research team were mindful of the potential for perceived anxiety among the participants regarding confidentiality issues in the study. Therefore, to mitigate this, during the data collection, I clarified my current status as a PhD student and explained the project and its purpose before conducting the interviews. Throughout the data collection process, I also noted if and how my interaction affected the participants and their responses. I reflected on this aspect after every interview to better position myself in the subsequent ones.

My background and experience in public health led participants from ministries other than MoHP to perceive me as a representative of MoHP. Aware of this dynamic, I made a deliberate effort to stay neutral during the data collection process and reemphasized my position as a student aiming to gain a deeper understanding of climate change and health equity policy landscape in Nepal. This however, also proved to be advantageous, as they valued the opportunity to discuss the issue and put forward the actions that they thought MoHP should be taking to advance climate change and health issue in Nepal. Additionally, participants from MoHP viewed me as a colleague and were more forthcoming with the issues, enabling discussions transparently on a number of areas, which otherwise could have been difficult to mention. At a personal level, my prior experience in the country made me feel at ease in most of the interviews even while interviewing high level officials. For me, many interviews seemed more like general conversation rather than a formal audio recorded interview, which facilitated the data collection process.

I recently conducted a content analysis of climate change and health policies in Nepal to examine the extent of health equity integration in climate change-related policies. The process and findings from this study helped me better prepare for the interviews as a researcher and allowed me to ask additional probing questions to gain deeper insights. However, while doing so, I was careful about not presenting the data collection as an interrogation to the participants and subtly asking the follow up questions for data triangulation, in case of major discrepancies noted.

Throughout the study, while I was consistently conscious of my role as a researcher and the potential influence my familiarity and knowledge about the policy making culture in Nepal could have on the way I see different aspects of the data, one of the other co-authors (SCB), is also an active member of the current policy landscape in Nepal. Therefore, in order to avoid any biases, he was not involved during the data collection or the initial analysis of the study. The research team members also took care to ensure that their familiarity and expertise in the subject did not influence the interpretation of the findings. For this reason, the data coding and interpretation process were continuously shared and discussed with the third author (MB), who relatively has little involvement in the national processes and was in a position to provide an unbiased perspective.
